# Why mild contrast medium-induced reactions are sometimes over-treated and moderate/severe reactions of internal organs are undertreated: a summary based on RadioComics

**DOI:** 10.1186/s13244-023-01554-y

**Published:** 2023-11-19

**Authors:** Paolo Lombardo, Knud Nairz, Ingrid Boehm

**Affiliations:** Department of Diagnostic, Interventional, and Pediatric Radiology, University of Bern, Academic Hospital (Inselspital), Bern, Switzerland

**Keywords:** Contrast-medium induced adverse reactions, Prophylaxis, Remedications, Renal complaints, Hypersensitivity reaction

Patients with underlying conditions, such as allergic predisposition or specifically contrast medium-allergy are at increased risk to acquire an adverse drug reaction upon re-exposure to contrast materials, and therefore should undergo a prophylactic management before receiving contrast medium (CM) [1, 2]. Interestingly, patients with minor and harmless complaints following CM injection, such as erythema, regularly receive a maximal pre-treatment (e.g., prednisolone plus H1-antagonist) [3] (Fig. 1). On the other hand, patients with reduced kidney function, renal insufficiency, and nephrotoxic medication should also undergo special prophylaxis protocols, such as naïve examination (without CM), low-dose CM application, or will be referred to an alternative modality [4], especially when multiple CM-applications during the last 2–3 days have been given. The experience shows that such patients are rarely candidates for a prophylaxis (Fig. 1). Therefore, the objective of this paper is to draw attention to the phenomenon that patients with a history of mild cutaneous CM reactions are subjected more frequently and intensively to premedication than patients with moderate to severe systemic reactions.

It appears to be counterintuitive that more severe disease patterns are associated with a less stringent premedication protocol and vice versa. We did not find a study in the literature to address this dissent. Therefore, we speculate that visually apparent allergic symptoms after CM injection are more likely to trigger an appropriate, or sometimes even overcautious response and pre-treatment when patients are re-admitted than a, e.g., further impairment of the damaged kidneys, which is non-immediate and therefore is very likely to remain unnoticed by the radiologist.

It has been demonstrated previously that so-called *RadioComics* [5] can serve as an artistic complement to more evidence-based methods like Radiomics to boil complex issues down. Premedication is stressful or problematic for the patients. For example, H1 antagonists (antihistamines) have a sedative effect. Outpatients are therefore not allowed to drive a car following the examination. Corticosteroids are problematic, for example, in patients with infectious diseases, osteoporosis, and diabetes mellitus.

Therefore, radiologists should adjust their prophylactic regimen according to patients’ preferences [6], and underlying diseases (Table 1), respectively. In other words, in patients at risk prophylaxis is necessary, but with respect to anti-allergy drugs one should realize that in most cases less is more [2, 3, 7]. Possibly, artificial intelligence will help to reduce the dose of contrast-enhanced CT (CECT) examinations, the number of CECTs [8], and to find the optimal individual prophylaxis.

**Table 1:** Recommended procedure dependent on selected previous adverse reactions

History of…	Suggested prophylaxis
**…mild reactions**	
Heat/cold feeling Erythema, flush Itching Localized urticaria Nausea/vomiting Dizziness	• Application of a non-culprit CM • Injection of low-dose CM (with reduced injection speed) • (none)
**…moderate or severe reactions**	
Contrast-induced acute kidney injury (CI-AKI)	• Prophylaxis (e.g., hydration) adapted to eGFR/serum-creatinine [9] • Consideration of nephrotoxic drugs • Consideration of several contrast-enhanced imaged guided examinations within a few days
Generalized urticaria Angioedema Conjunctivitis, rhinitis Hypo-/hypertension Mild bronchial symptoms Anaphylaxis	Application of a non-culprit CM according to the results of an allergy skin testing

**Fig. 1:**
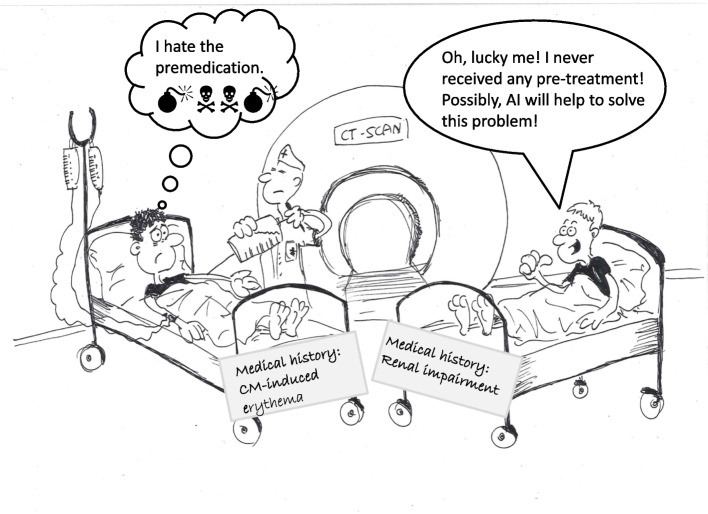
Patient with a history of a mild adverse reaction in the past receives a premedication (left). While the patient with a history of moderate or severe adverse reaction receives no prophylaxis (right)

Here we present a new *RadioComic* that seizes this topic ironically.

## Data Availability

Not applicable.

## Abbreviations

CECT: Contrast-enhanced computed tomography
CM: Contrast medium
